# Investigation of Non-Covalent Interactions of Aflatoxins (B1, B2, G1, G2, and M1) with Serum Albumin

**DOI:** 10.3390/toxins9110339

**Published:** 2017-10-25

**Authors:** Miklós Poór, Mónika Bálint, Csaba Hetényi, Beatrix Gődér, Sándor Kunsági-Máté, Tamás Kőszegi, Beáta Lemli

**Affiliations:** 1 Department of Pharmacology, Faculty of Pharmacy, University of Pécs, Szigeti út 12, Pécs H-7624, Hungary; goderbea@freemail.hu; 2 Department of Pharmacology and Pharmacotherapy, Medical School, University of Pécs, Szigeti út 12, Pécs H-7624, Hungary; monibalint18@gmail.com (M.B.); csabahete@yahoo.com (C.H.); 3 Department of General and Physical Chemistry, University of Pécs, Ifjúság útja 6, Pécs H-7624, Hungary; kunsagi@gamma.ttk.pte.hu (S.K.-M.); blemli@gamma.ttk.pte.hu (B.L.); 4 János Szentágothai Research Center, Ifjúság útja 20, Pécs H-7624, Hungary; koszegi.tamas@pte.hu; 5 Department of Laboratory Medicine, University of Pécs, Ifjúság útja 13, Pécs H-7624, Hungary

**Keywords:** aflatoxin B1, aflatoxins, serum albumin, albumin-ligand interaction, fluorescence spectroscopy

## Abstract

Aflatoxins are widely spread mycotoxins produced mainly by *Aspergillus* species. Consumption of aflatoxin-contaminated foods and drinks causes serious health risks for people worldwide. It is well-known that the reactive epoxide metabolite of aflatoxin B1 (AFB1) forms covalent adducts with serum albumin. However, non-covalent interactions of aflatoxins with human serum albumin (HSA) are poorly characterized. Thus, in this study the complex formation of aflatoxins was examined with HSA applying spectroscopic and molecular modelling studies. Our results demonstrate that aflatoxins form stable complexes with HSA as reflected by binding constants between 2.1 × 10^4^ and 4.5 × 10^4^ dm^3^/mol. A binding free energy value of −26.90 kJ mol^−1^ suggests a spontaneous binding process between AFB1 and HSA at room-temperature, while the positive entropy change of 55.1 JK^−1^ mol^−1^ indicates a partial decomposition of the solvation shells of the interacting molecules. Modeling studies and investigations with site markers suggest that Sudlow’s Site I of subdomain IIA is the high affinity binding site of aflatoxins on HSA. Interaction of AFB1 with bovine, porcine, and rat serum albumins was also investigated. Similar stabilities of the examined AFB1-albumin complexes were observed suggesting the low species differences of the albumin-binding of aflatoxins.

## 1. Introduction

**Figure 1:**
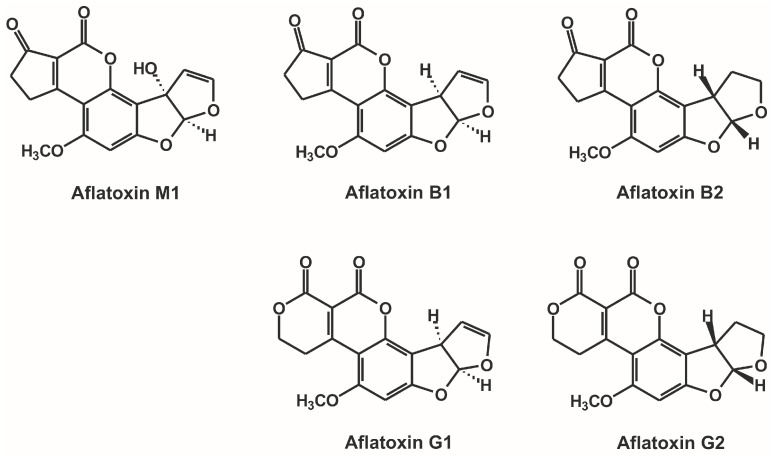
Chemical structures of the examined aflatoxins.

Mycotoxins are secondary metabolic products of filamentous fungi causing toxic actions in the body of animals and humans [1]. Four aflatoxins occur naturally (Figure 1), these are aflatoxin B1 (AFB1), B2 (AFB2), G1 (AFG1), and G2 (AFG2); AFB2 and AFG2 are barely toxic unless they are oxidized to AFB1 and AFG1 in vivo [2]. Aflatoxins are found in different grains or foodstuffs (e.g., corn, peanuts, sorghum, and rice) causing serious health risks for people worldwide [3,4,5]. AFB1 is one of the best-known, widely spread mycotoxin produced mainly by *Aspergillus flavus*. The main target organ of AFB1 is the liver causing hepatocellular carcinoma; however, other toxic effects are also attributed to AFB1, including lethal acute intoxication through highly contaminated food [2]. AFB1 has been classified as a Group I carcinogen by the International Agency for Research on Cancer (IARC) [6]. After the biotransformation of AFB1 by cytochrome P450 (CYP) enzymes (e.g., CYP1A2 and CYP3A4 in humans), a reactive epoxide metabolite of AFB1 (aflatoxin-8,9-epoxide) is produced [7,8]. Aflatoxin-8,9-epoxide is able to form covalent adducts with DNA (N7-guanine adduct) in tissues as well as with serum albumin (lysine adducts) in the circulation [9,10]. Furthermore, through the hepatic hydroxylation of AFB1, its major metabolic product, aflatoxin M1 (AFM1) is formed, which is present in urine as well as in human breast milk [2,11,12]. AFM1 is the most frequent aflatoxin in animal milk therefore it commonly occurs in several dairy products [11].

Human serum albumin (HSA) is the most abundant plasma protein in the human circulation. HSA is a highly important molecule in the human organism: HSA maintains the oncotic pressure and the pH in the human blood; it possesses antioxidant and pseudo-enzymatic properties and furthermore, HSA is able to form stable non-covalent complexes with several compounds resulting in their albumin-bound transport in the circulation [13]. Therefore, albumin-ligand interaction can play an important role regarding the tissue distribution and elimination of the bound ligand molecules [14]. HSA is built up by three domains (I, II, and III) and each domain contains two subdomains (A and B). There are three major binding sites of drugs and xenobiotics on HSA: Sudlow’s Site I (or drug binding Site I; on subdomain IIA), Sudlow’s Site II (or drug binding Site II; on subdomain IIIA), and Heme binding site (on subdomain IB) [13,15]. Previous studies highlighted that some mycotoxins (e.g., citrinin, deoxynivalenol, ochratoxin A, patulin, and zearalenone) are able to form stable non-covalent complexes with HSA [16,17,18,19,20]. Covalent interaction of the epoxide metabolite of AFB1 is well-known, and can be used as a marker of AFB1 exposition [21,22]. Despite the non-covalent AFB1-albumin interaction was reported by Dirr and Schabort [23], many details are unclear including the binding site of AFB1 on HSA. Furthermore, the interactions of other aflatoxins (e.g., AFB2, AFG1, AFG2, and AFM1) with serum albumin were not examined previously. Investigation of protein-ligand interactions can be performed by several techniques including equilibrium dialysis, ultrafiltration, chromatography, capillary electrophoresis, spectroscopy (UV-Vis, fluorescence, IR, Raman, circular dichroism, etc.), calorimetry, surface plasmon resonance, and so on [24]. Today, many new, more sensitive, and sophisticated techniques have been discovered and used for the analytical detection of aflatoxins as well as for the investigation of the molecular interactions of these mycotoxins, including mass spectrometry (MS) and surface enhanced Raman scattering (SERS) [25]. Aptamers and aptasensors are also commonly applied tools used for recognition and analytics of aflatoxins and other mycotoxins [26,27]. Regarding albumin-ligand interactions, fluorescence spectroscopy is a relatively cheap and sensitive, widely accepted, and commonly applied technique which is suitable to investigate binding strengths and binding site(s) of the formed complexes [28,29,30,31]. Furthermore, the results of fluorescence spectroscopic investigations can be supported by other analytical techniques or with molecular modeling studies which also contributes to the deeper understanding of the complex formation and promote the localization of binding site(s) [16,20].

In this study, the interaction of aflatoxins (AFB1, AFB2, AFG1, AFG2, and AFM1) with HSA was investigated using fluorescence spectroscopic and molecular modelling studies. Spectral changes of AFB1 in the presence of HSA as well as fluorescence quenching of HSA by aflatoxins were examined. To better understand the complex formation of AFB1 with HSA, thermodynamic studies were performed as well. Primary binding sites of aflatoxins on HSA were identified by applying site markers and structural calculations. Finally, to investigate the potential species differences of AFB1-albumin interaction, complex formation of AFB1 was tested with bovine, porcine, and rat serum albumins as well.

## 2. Results and Discussion

### 2.1. Fluorescence Properties of AFB1 in the Absence and Presence of HSA

**Figure 2:**
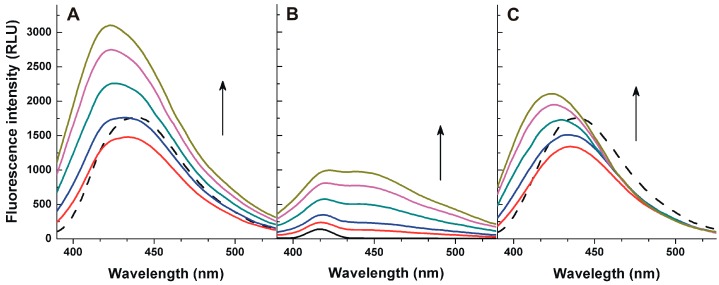
Fluorescence emission spectra of 1 μM AFB1 in the absence (**A**, dotted line) and in the presence of increasing HSA concentrations (**A**, solid lines; 2.5, 5.0, 10, 15, and 20 μM). Fluorescence emission spectra of increasing HSA concentrations in the absence of AFB1 (**B**; 2.5, 5.0, 10, 15, and 20 μM). The subtraction of the spectra represented in (**A**) (*I_AFB1+HSA_*) and (**B**) (*I_HSA_*) is demonstrated in (**C**) (*I_AFB1+HAS_–I_HSA_*) (λ_exc_ = 365 nm; PBS, pH 7.4).

First, fluorescence spectroscopic properties of AFB1 were investigated in the absence and presence of HSA, in PBS (pH 7.4). AFB1 showed its fluorescence excitation and emission maxima at 365 and 440 nm, respectively. In the presence of increasing concentrations of HSA, significant changes of the fluorescence emission spectra of AFB1 were observed (Figure 2A). Using 365 nm as excitation wavelength, both AFB1 (1 μM; Figure 2A, indicated with dashed line) and the HSA preparation (2.5–20 μM; Figure 2B) gave fluorescence emission signal at 440 nm, suggesting that the increased fluorescence signal is partly derived from the fluorescence emission of the HSA preparation. On the other hand, after the subtraction of the fluorescence spectra of HSA from the combined spectra of AFB1 and HSA (*I_AFB1+HAS_–I_HSA_*), we noticed that the emission maximum of AFB1 shows a blue shift, and significant changes of the fluorescence emission intensities were observed as well (Figure 2C). Based on these results, it is reasonable to hypothesize that the spectral changes resulted from the complex formation of AFB1 with HSA.

### 2.2. Investigation of Aflatoxin-HSA Interactions by Fluorescence Quenching Method

**Figure 3:**
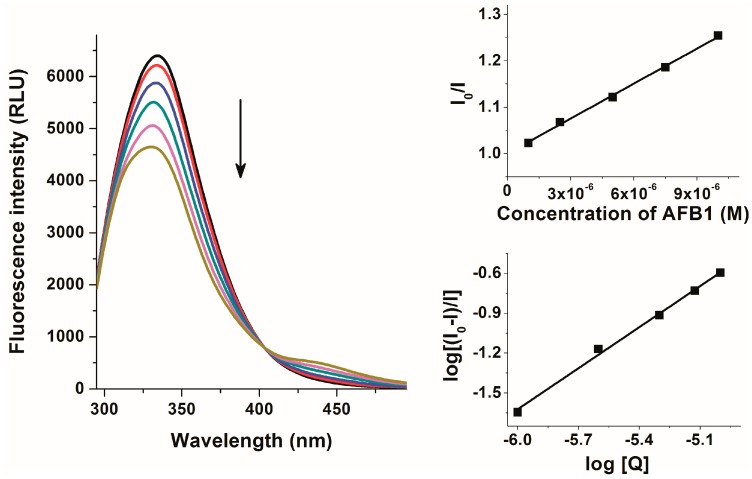
Fluorescence emission spectra of 2 μM HSA in the absence and presence of increasing AFB1 concentrations (1.0, 2.5, 5.0, 7.5, and 10 μM) in PBS at pH 7.4 (**left**) as well as Stern-Volmer (**top right**) and double logarithmic Stern-Volmer (**bottom right**) plots of the interaction (λ_exc_ = 280 nm, λ_em_ = 334 nm).

In order to characterize aflatoxin-HSA interactions, fluorescence quenching studies were performed. Increasing aflatoxin concentrations (0–10 μM) were added to a standard amount of HSA (2 μM) then fluorescence emission spectra of HSA were recorded (λ_exc_ = 280 nm). Aflatoxins alone did not give fluorescence signal at the emission maximum of HSA (334 nm) under the applied conditions. To exclude the possible involvement of the inner filter effect, fluorescence emission intensities were corrected based on the absorbance of aflatoxins (Equation (1)). Even the presence of 1 μM aflatoxin concentrations led to the significant decrease of the fluorescence emission intensity of HSA at 334 nm, and increasing aflatoxin concentrations caused further reduction of the emission signal (Figure 3). The significant quenching of the Trp-214 moiety of HSA by aflatoxins suggests that the binding site of aflatoxins on HSA needs to be located close to the Trp-214 amino acid, suggesting Sudlow’s Site I as a potential binding site.

The graphical application of the Stern-Volmer (Equation (2)) and double logarithmic Stern-Volmer (Equation (3)) equations showed good correlation with the 1:1 model of the complex formation. In agreement with these results, non-linear fitting by Hyperquad2006 program (Equation (4)) suggested 1:1 stoichiometry as well. Decimal logarithmic values of *K_SV_*, *K_a_*, and *K* are demonstrated in Table 1. Although log*K* values were slightly higher compared to log*K_SV_* and log*K_a_*, each calculation indicated similar stabilities, and agreed that the affinities of aflatoxins toward HSA are almost the same (only AFG2-HSA complex showed slightly lower stability). These data suggest that aflatoxins form stable non-covalent complexes with HSA. The determined binding constants of AFB1-HSA complex is consistent with the previously reported data of Dirr and Schabort [23]. Furthermore, stabilities of aflatoxin-HSA complexes are comparable with deoxynivalenol-HSA and patulin-HSA complexes [17,18]. On the other hand, some mycotoxins including citrinin, zearalenone, and mainly ochratoxin A form considerably stronger complexes with HSA than aflatoxins [16,19,20]. Based on the calculated data, it is reasonable to hypothesize that the complex formations of aflatoxins with HSA are probably biologically relevant interactions.

### 2.3. Thermodynamic Studies

In order to investigate the temperature dependence of AFB1-HSA interaction, binding constants were determined between 25 and 40 °C. Results show higher stability of the complexes at lower temperatures reflecting formation of ground state complexes. Using the temperature-dependence of the stability constants, the thermodynamic parameters associated to the interaction of AFB1 with HSA was also determined using the van’t Hoff equation (Equation (5)). The calculated negative Δ*G* value (T = 298.16 K, −26.90 kJ mol^−1^) suggests the spontaneous binding process between AFB1 and HSA at room-temperature. This value is within the typical range of non-covalent interactions. Δ*H* and Δ*S* values of the AFB1-HSA complex formation were −10.48 kJ mol^−1^ and 55.1 JK^−1^ mol^−1^, respectively. Comparing these values with the parameters obtained for previously examined mycotoxin-albumin complexes, namely citrinin-HSA and zearalenone-HSA (Table 1), we can conclude that the smaller enthalpy change is associated with higher entropy gain in agreement with the enthalpy-entropy compensation. The positive value of the entropy change supposes the decomposition of the solvation shells of the interacting molecules (or a part of them) leading to less ordered structure of water molecules and suggest hydrophobic property of binding site of HSA for AFB1 molecules. Considering that the stability constant of the AFB1-HSA interaction is very close to the zearalenone-HSA interaction at room-temperature, the difference between the entropy gains of the two reactions reflects that the AFB1 molecules lose and the zearalenone molecules keep their solvation shell during the interaction with HSA. These results are consistent with the potential involvement of Sudlow’s Site I as high affinity binding site of AFB1 on HSA.

### 2.4. Molecular Modeling of the Binding of Aflatoxins to HSA

**Figure 4:**
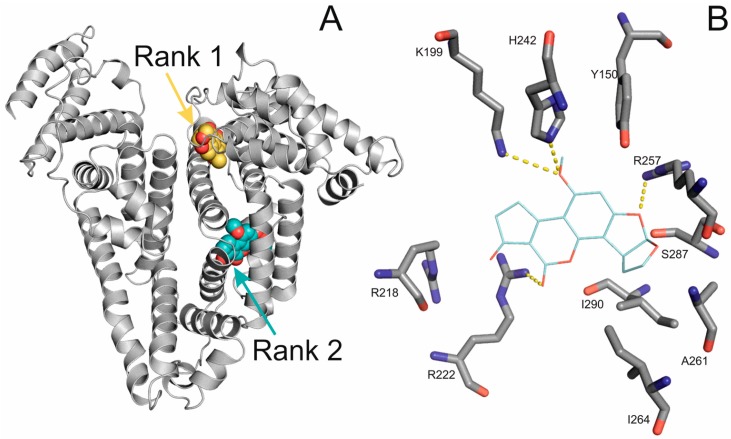
HSA is represented with grey cartoon, and AFB1 conformation from the first rank is represented by yellow spheres, and AFB1 from the second rank is represented with cyan spheres (**A**); AFB1 is represented with cyan, thin sticks, and the interacting amino acids are represented with grey thick sticks (**B**).

A blind docking (BD) search was performed on the entire surface of HSA for the binding site(s) of the herein investigated molecules. Ranking of the binding positions was performed by the binding energy calculated with AutoDock 4 scoring function. In all of the five investigated molecules, after BD calculations, the first rank representative was bound to the Heme binding site (Figure 4A, yellow spheres), while the second rank (the fourth rank in case of AFG2, Table 2) found Sudlow’s site I (Figure 4A, cyan spheres). As it is shown in Figure 4B, AFB1 is fixed in the pocket of Sudlow’s site I by salt bridges with R222 and R257, and H-bonds with H242 and K199. The hydrophilic interactions are fixating the ligand from both directions, but hydrophilic interactions with I290, A261 and I264 can also be noticed.

In the case of AFB1 complexed with Sudlow’s site I, a remarkable binding free energy of −32.05 kJ mol^−1^ was calculated, which is comparable to those of the metabolites (Table 3). The metabolites of AFB1, were docked to the Sudlow’s site I with focused docking, and the corresponding relative calculated binding free energies are listed in Table 3, as well. The differences in binding energies between AFB1 and its metabolites are within the range of calculation error. Accordingly, the binding modes of the metabolites are similar to that of AFB1 presented in Figure 4. The above modeling results are in accordance with the fluorescence studies presented in Table 1 and provide the atomic resolution structural background of the binding of the investigated ligands (Figure 1) to HSA.

### 2.5. Investigation of the Binding Site of Aflatoxins on HSA Using Site Markers

One of the binding sites suggested by modeling studies is Sudlow’s Site I (subdomain IIA) which is in good agreement with our previous hypothesis based on the fact that aflatoxins were able to significantly decrease Trp-214 fluorescence, even in the presence of their low concentrations. To test this idea, the influence of AFB1 on warfarin-HSA interaction was investigated. Warfarin (WAR) is the most commonly applied site marker of Sudlow’s Site I. During this experiment, our previously published model was applied, based on the observation that the fluorescence signal of WAR significantly increases after the complex formation with albumin [16,31]. Because the fluorescence enhancement of WAR by HSA results in approximately 20-fold higher fluorescence signal of HSA-bound WAR compared to free WAR (λ_exc_ = 317 nm, λ_em_ = 379 nm), displacement of WAR from HSA leads to the strong decrease of its fluorescence [31]. 

**Figure 5:**
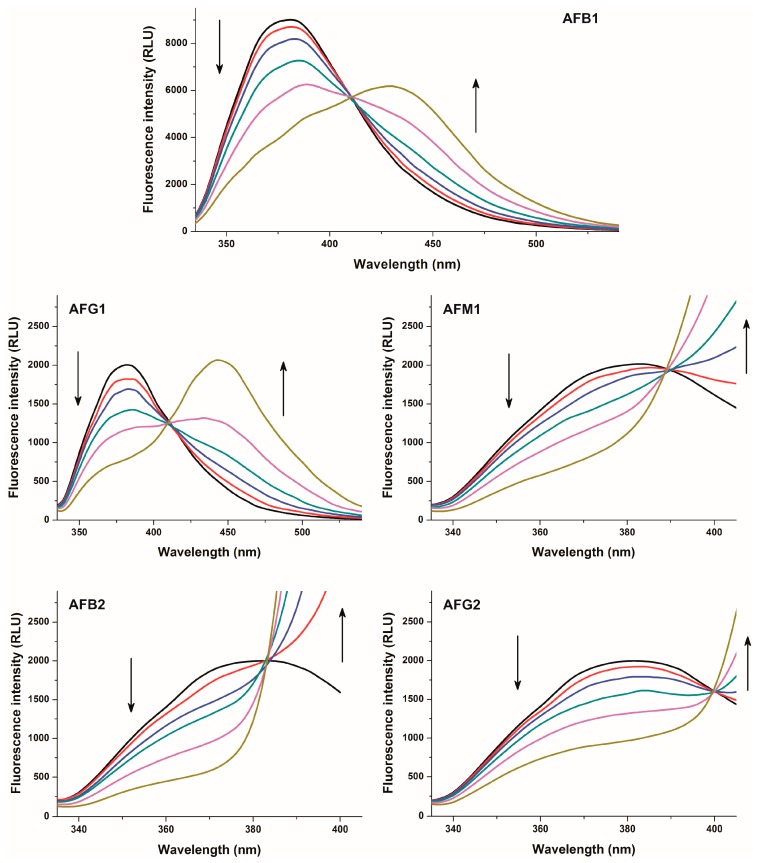
Fluorescence emission spectra of 1 μM warfarin in the presence of 3.5 μM HSA, and in the absence and presence of increasing aflatoxin concentrations (0.0 μM: black, 1.0 μM: red, 2.5 μM: blue, 5.0 μM: green, 10 μM: purple, and 20 μM: brown) in PBS (λ_exc_ = 317 nm).

To test the effect of aflatoxins on the albumin binding of WAR, increasing concentrations of aflatoxins (1.0, 2.5, 5.0, 10, and 20 μM) were added to 1 μM WAR and 3.5 μM HSA. Fluorescence emission spectra were recorded using 317 nm as excitation wavelength (the excitation maximum of HSA-bound WAR). As Figure 5 demonstrates, the presence of AFB1 led to the significant decrease of the fluorescence signal at 379 nm indicating the displacement of WAR from HSA. Furthermore, the red shift of the spectra and the appearance of a peak at 440 nm resulted from the fluorescence signal expressed by AFB1 itself. Very similar spectroscopic changes were observed in the presence of increasing concentrations of AFG1. Despite the fact that other aflatoxins (AFM1, AFB2, and AFG2) express much stronger fluorescence than AFB1 and AFG1 (therefore they give fluoresce signal even at 379 nm under the applied circumstances), these mycotoxins also considerably decreased the fluorescence intensity of WAR at 379 nm (Figure 5). These results highlight that aflatoxins are able to significantly displace warfarin from HSA and strongly suggest that Sudlow’s Site I is the high affinity binding site of aflatoxins on HSA. Moreover, Figure 5 also demonstrates that only relatively high aflatoxin concentrations resulted in the marked displacement of WAR from HSA. Assuming the competitive nature of the displacement of WAR from HSA by aflatoxins, this observation suggests that aflatoxins bind with significantly lower affinity to HSA than WAR. This observation is in good agreement with our previous results, because the calculated log*K* values of aflatoxin-HSA complexes (Table 1) are significantly lower compared to the WAR-HSA complex (log*K* = 5.3) [31]. 

Another potential binding site that was suggested by modeling studies was subdomain IB (Heme binding site). To test the involvement of this binding site, the influence of aflatoxins on the albumin binding of methyl orange (MO) was examined. Bilirubin and biliverdin are the typically applied ligands of this binding site, however, these compounds bind to albumin with extremely high affinity [32], while the binding constant of MO-HSA complex (log*K* ~ 5) is comparable with aflatoxin-HSA complexes [15]. Complex formation of MO with HSA results in a red shift of its absorption maximum (464 → 477 nm) and an increase of its absorbance (Figure S1). HSA or aflatoxins alone or in combination showed no absorbance at the wavelength maxima of MO or MO-HSA complex under the applied circumstances. In order to investigate the effect of aflatoxins on MO-HSA interaction, increasing concentrations of aflatoxins (5–50 µM) were added to 10 µM MO and 15 µM HSA. Aflatoxins had no effect on the absorption spectrum of MO-HSA complex suggesting the negligible involvement of subdomain IB as a high affinity binding site of aflatoxins.

### 2.6. Interaction of AFB1 with Bovine, Porcine, and Rat Serum Albumins

Stabilities of mycotoxin-albumin complexes show marked species differences in some cases (e.g., ochratoxin A) [19]. For this reason, the interaction of AFB1 was investigated with bovine (BSA), porcine (PSA), and rat (RSA) serum albumins as well. In order to quantify the stabilities of these AFB1-albumin complexes, fluorescence quenching studies were performed. Increasing AFB1 concentrations (0–10 μM) were added to 2 μM albumin and fluorescence emission spectra were recorded (λ_exc_ = 280 nm). Thereafter, *K_SV_*, *K_a_*, and *K* values were quantified as described in Section 2.2. As Table 4 demonstrates, AFB1 binds to HSA, BSA, and PSA with the same affinities, while the stability of AFB1-RSA complex is approximately 2-fold higher compared to the other examined AFB1-albumin complexes. These results highlight that the affinities of AFB1 toward different albumin species are very similar; only AFB1-RSA complex showed somewhat higher stability but the magnitude of its binding constant is the same.

## 3. Conclusions

In this study, the non-covalent interactions of aflatoxins with serum albumins were investigated using spectroscopic and molecular modeling studies. Our results demonstrate that aflatoxins form stable complexes with HSA occupying Sudlow’s Site I as high affinity binding site. AFB1, AFB2, AFG1, and AFM1 bind to HSA with very similar affinity, while the stability of AFG2-HSA complex is slightly lower compared to the other examined aflatoxin-albumin complexes. The complexes of AFB1 with human, bovine, porcine, and rat albumins showed similar stabilities. Based on these observations, the results of animal experiments with rats or pigs regarding the non-covalent albumin binding of AFB1 may be extrapolated to humans. Furthermore, BSA is commonly applied to study albumin-ligand interactions, and BSA seems suitable to model AFB1-albumin complex formation because BSA and HSA bind to AFB1 with very similar affinities. Based on our pharmacological knowledge regarding plasma protein binding of different drugs, the determined binding constants of aflatoxin-albumin complexes (10^4^–10^5^ dm^3^/mol) are in the range of biologically relevant interactions, suggesting the potential toxicological importance of the albumin binding of aflatoxins. Aflatoxins are non-ionized molecules, therefore, they are able to pass through the cell membrane by passive diffusion. Because the significant albumin binding results in the low concentrations of free aflatoxins (which is the driving force of their passive diffusion), it can slow down the tissue distribution of aflatoxins (including their uptake into the liver). The strong albumin-binding may also lead to the relatively slower elimination of aflatoxins based on the same principles. Similarly to aflatoxins, many other compounds occupy Site I on albumins, so displacement of aflatoxins from albumin and the toxicological consequences of this process is a very interesting issue as well. Displacement can cause increased toxicity (because of the elevated tissue uptake) or can even alleviate the toxicity of aflatoxins (if their elimination is faster compared to their cellular uptake). Therefore, further investigation of aflatoxin-albumin interactions is reasonable with the involvement of animal studies, because in vivo experiments are necessary to clearly answer these questions.

## 4. Materials and Methods

### 4.1. Reagents

All of the applied reagents and solvents were of spectroscopic or analytical grade. Aflatoxin B1 (AFB1), aflatoxin B2 (AFB2), aflatoxin G1 (AFG1), aflatoxin G2 (AFG2), human serum albumin (HSA), bovine serum albumin (BSA), porcine serum albumin (PSA), rat serum albumin (RSA), and warfarin (WAR) were obtained from Sigma-Aldrich. Aflatoxin M1 (AFM1) was purchased from Apollo Scientific, while Methyl orange (MO) was from Reanal. 2000 μM stock solution of aflatoxins was prepared in dimethyl sulfoxide (Fluka, spectroscopic grade) and stored at −20 °C. To mimic the extracellular physiological conditions, phosphate buffered saline (PBS, pH 7.4) was applied as solvent during our measurements.

### 4.2. Spectroscopic Measurements

Fluorescence measurements were performed employing Hitachi F-4500 fluorescence spectrophotometer. UV-Vis spectra were recorded applying Specord Plus 210 (Analytic Jena AG, Jena, Germany) spectrophotometer. All measurements were carried out at 25 °C.

During fluorescence quenching experiments, emission intensities of albumin were determined in the presence of 2 μM albumin with and without aflatoxins (0–10 μM) using 280 and 334 nm as excitation and emission wavelengths, respectively. Inner filter effects of aflatoxins were eliminated applying the following equation [33]:

(1) $$I_{cor}=I_{obs}\times e^{(Aexc+Aem)/2}$$

where *I_cor_* and *I_obs_* are the corrected and observed fluorescence emission intensities, respectively; while *A_exc_* and *A_em_* are the absorption of aflatoxins at 280 and 334 nm, respectively.

Complex formations of aflatoxins with albumins were investigated applying the Stern-Volmer equation:

(2) $$\frac{I_{0}}{I}=1+K_{SV}\times [Q]$$

where *I* and *I*_0_ denote the fluorescence intensities of albumin with and without aflatoxins, respectively. *K_SV_* (with the unit of dm^3^/mol) is the Stern-Volmer quenching constant and [*Q*] is the molar concentration of aflatoxins.

Association constants (*K_a_*) and number of binding sites (*n*) were determined by linear fitting, employing the double logarithmic Stern-Volmer equation [33]:

(3) $$\log\frac{\left(I_{0}-I\right)}{I}=\log K_{a}+n\times \log[Q]$$

where *I* and *I*_0_ denote the fluorescence intensities of albumin with and without aflatoxins, respectively; while [*Q*] is the molar concentration of aflatoxins.

Thereafter, binding constants of aflatoxin-albumin complexes were quantified by Hyperquad2006 program package, assuming 1:1 stoichiometry [29,30],

(4) $$I=I_{0}+\frac{\left(I_{HG}-I_{0}\right)}{2\cdot {\left[H\right]}_{0}}\cdot \left({\left[H\right]}_{0}+{\left[G\right]}_{0}+\frac{1}{K}-\sqrt{{\left({\left[H\right]}_{0}+{\left[G\right]}_{0}+\frac{1}{K}\right)}^{2}-4\cdot {\left[H\right]}_{0}\cdot {\left[G\right]}_{0}}\right)$$

where, *I*_0_ and *I* denote the emission intensities of albumin at 334 nm in the absence and presence of aflatoxins, respectively; *I_HG_* is the emission intensity of pure aflatoxin-albumin complex at 334 nm (calculated by the Hyperquad2006); *K* denotes the binding constant (with the unit of dm^3^/mol); [*H*]_0_ and [*G*]_0_ are the total concentrations of albumin and aflatoxin, respectively.

### 4.3. Thermodynamic Studies

Fluorolog τ3 spectrofluorometric system (Jobin-Yvon/SPEX) was applied during thermodynamic studies using 280 nm excitation wavelength. For data collection, a photon counting method with 0.1 s integration time was used. Excitation and emission bandwidths were set to 4 nm. A 10 mm layer thickness of the fluorescent probes with right angle detection was applied. The measurements were carried out at six different temperatures 25–40 °C using 3 °C steps. The binding constants were determined by the Hyperquad2006 program package (Equation (4)). The enthalpy and entropy changes of complex formation between AFB1 and HSA molecules were calculated as follows: the logarithms of binding constants (log*K*) at different temperatures were plotted against the reciprocal temperature according to the van’t Hoff equation. The enthalpy change and entropy change were derived from the slope and from the intercept of the fitted line, respectively:

(5) $$\log K=-\frac{\Delta G}{2.303RT}=-\frac{\Delta H}{2.303RT}+\frac{\Delta S}{2.303R}$$

where Δ*G* is the Gibbs free energy change, while Δ*H* and Δ*S* reflect the enthalpy and entropy changes of the association reaction, respectively. *R* is the gas constant and *T* refers the temperature.

### 4.4. Structural Calculations

Blind docking calculations were performed using the AutoDock 4.2 program package. Ligand molecules were built in Maestro. Energy-minimization of molecules was performed by the semi-empirical quantum chemistry program package, MOPAC. The geometries were optimized at a 0.001 gradient norm and subjected to subsequent force calculations using PM3 parameterization. In all cases, the force constant matrices were positive definite. The apo structure of HSA (pdb code 1a06) was used as a target of blind docking [34,35]. Gasteiger-Marsilli partial charges were added to both ligand and target atoms and a Kollman united atom representation was applied for groups with non-polar bonds. A Lamarckian genetic algorithm was used for search. For BD, the grid box was centered on the center of mass of the target. A grid map with a box size of 250 × 250 × 250 points and 0.375 Å spacing was calculated by AutoGrid 4. In the case of focused docking calculations on Sudlow’s site I, the grid box of 90 × 90 × 90, was centered on 35.000 31.825 37.000 coordinates. In all calculations, the number of docking runs was set to 100, numbers of energy evaluations and generations were 20 million. Ligand conformations that resulted from the docking runs were ordered by the corresponding calculated Δ*G* values and clustered using a tolerance of 1.75 Å distance between cluster members. Conformations with the lowest binding energy within a cluster were selected as cluster representatives.

## Figures and Tables

**Table 1:** Decimal logarithmic values of Stern-Volmer quenching constants (*K_SV_*; based on Equation (2)), association constants (*K_a_*), and number of binding sites (*n*; both based on Equation (3)), and binding constants (*K*; based on Equation (4)) of aflatoxin-HSA complexes (±SD).

	log*K_SV_*	log*K_a_*	*n*	log*K*
**AFB1–HSA**	4.44 ± 0.06	4.46 ± 0.08	1.005 ± 0.029	4.65 ± 0.01
**AFB2–HSA**	4.21 ± 0.02	4.40 ± 0.08	1.054 ± 0.000	4.55 ± 0.01
**AFG1–HSA**	4.33 ± 0.04	4.43 ± 0.03	1.020 ± 0.019	4.58 ± 0.01
**AFG2–HSA**	3.96 ± 0.04	4.04 ± 0.07	1.017 ± 0.025	4.34 ± 0.01
**AFM1–HSA**	4.28 ± 0.01	4.26 ± 0.00	1.004 ± 0.013	4.52 ± 0.01

**Table 2:** Thermodynamic parameters associated to the formation of complexes of HSA with AFB1 and with two other mycotoxins.

Complex	Δ*H* kJ mol^−1^	Δ*S* JK^−1^ mol^−1^	Δ*G* (298.16 K) kJ mol^−1^
**AFB1-HSA ^1^**	−10.48	55.09	−26.90
**citrinin-HSA ^2^**	−24.15	20.90	−29.96
**zearalenone-HSA ^3^**	−30.09	−3.50	−29.06

^1^ present work; ^2^ previously reported data [16]; ^3^ previously reported data [20].

**Table 3:** Blind and focused docking of the ligands to HSA. Calculated binding free energies of the ligands bound to Sudlows’s site I are compared to that of AFB1 and listed as relative values (RBE). Thus, RBE of AFB1 is 1 by definition. The corresponding rank number (#Rank) of Sudlows’s site I is also shown.

Ligand	Blind Docking	Blind Docking	Focused Docking	Focused Docking
	RBE	#Rank	RBE	#Rank
**AFB1**	1	2	-	-
**AFB2**	0.98	2	0.99	1
**AFG1**	0.95	2	0.95	1
**AFG2**	0.98	4	0.99	1
**AFM1**	0.98	2	0.97	1

**Table 4:** Decimal logarithmic values of Stern-Volmer quenching constants (*K_SV_*; based on Equation (2)), association constants (*K_a_*) and number of binding sites (*n*; both based on Equation (3)), and binding constants (*K*; based on Equation (4)) of AFB1-albumin complexes (±SD).

	log*K_SV_*	log*K_a_*	*n*	log*K*
**AFB1–HSA**	4.44 ± 0.06	4.46 ± 0.08	1.005 ± 0.029	4.65 ± 0.01
**AFB1–BSA**	4.41 ± 0.06	4.44 ± 0.01	1.006 ± 0.015	4.63 ± 0.01
**AFB1–PSA**	4.45 ± 0.00	4.52 ± 0.10	1.023 ± 0.034	4.62 ± 0.01
**AFB1–RSA**	4.82 ± 0.01	4.90 ± 0.11	0.998 ± 0.051	4.91 ± 0.01

## Supplementary Materials

The following are available online at www.mdpi.com/2072-6651/9/11/339/s1, Figure S1: Absorption spectra of 10 μM methyl orange in the absence and presence of increasing HSA concentrations (5, 10, 15, 20, and 30 μM) in PBS.
