# Supplementary material for: Investigation of Non-Covalent Interactions of Aflatoxins (B1, B2, G1, G2, and M1) with Serum Albumin
Source: Toxins (Basel). 2017 Oct 25;9(11):339. doi: 10.3390/toxins9110339 (PMC5705954; doi:10.3390/toxins9110339)
Supplement: Supplementary file 1 [file toxins-09-00339-s001.pdf]

Supplementary Materials: Investigation of Non-Covalent Interactions of Aflatoxins (B1, B2, G1, G2, and M1) with Serum Albumin

Miklós Poór, Mónika Bálint, Csaba Hetényi, Beatrix Gődér, Sándor Kunsági-Máté, Tamás Kőszegi and Beáta Lemli

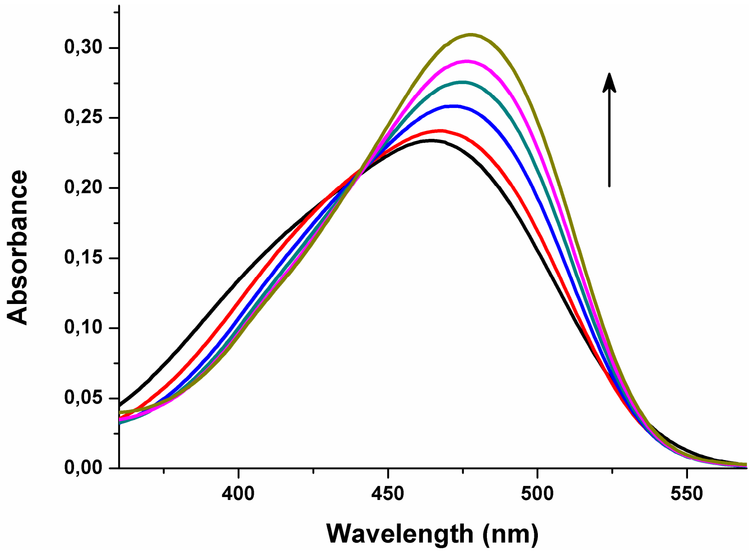

Figure S1. Absorption spectra of 10 μM methyl orange in the absence and presence of increasing HSA concentrations (5, 10, 15, 20, and 30 μM) in PBS.
